# Analysis of *Salmonella* lineage-specific traits upon cell sorting

**DOI:** 10.3389/fcimb.2023.1146070

**Published:** 2023-03-29

**Authors:** Rocío Fernández-Fernández, Rocío López-Igual, Josep Casadesús, María Antonia Sánchez-Romero

**Affiliations:** ^1^ Departamento de Genética, Facultad de Biología, Universidad de Sevilla, Seville, Spain; ^2^ Departamento de Microbiología y Parasitología, Facultad de Farmacia, Universidad de Sevilla, Seville, Spain; ^3^ Instituto de Bioquímica Vegetal y Fotosíntesis, Universidad de Sevilla and C.S.I.C., Seville, Spain

**Keywords:** cell sorting, bacterial lineages, phenotypic heterogeneity, flow cytometry, *Salmonella*

## Abstract

Microbial cell individuality is receiving increasing interest in the scientific community. Individual cells within clonal populations exhibit noticeable phenotypic heterogeneity. The advent of fluorescent protein technology and advances in single-cell analysis has revealed phenotypic cell variant in bacterial populations. This heterogeneity is evident in a wide range of phenotypes, for example, individual cells display variable degrees of gene expression and survival under selective conditions and stresses, and can exhibit differing propensities to host interactions. Last few years, numerous cell sorting approaches have been employed for resolving the properties of bacterial subpopulations. This review provides an overview of applications of cell sorting to analyze *Salmonella* lineage-specific traits, including bacterial evolution studies, gene expression analysis, response to diverse cellular stresses and characterization of diverse bacterial phenotypic variants.

## 1 Introduction

Bacterial cells with identical genotypes habitually display different phenotypes under identical conditions (Avery, 2006; Sánchez-Romero and Casadesús, 2021b). This heterogeneity is manifest in a wide range of phenotypes, displaying variability on gene expression, bacterial differentiation, survival under selective conditions and stresses, and pathogen-host interactions (Dhar and McKinney, 2007; Stewart et al., 2011; Ceuppens et al., 2013; Sánchez-Romero and Casadesús, 2018). Formation of bacterial lineages can make adaptive value and may facilitate bacterial adaptation to hostile and/or unpredictable environments (van der Woude and Baumler, 2004; Dubnau and Losick, 2006; Casadesús and Low, 2013; Sánchez-Romero and Casadesús, 2020). Phenotypic heterogeneity is an extensive phenomenon in many bacterial species including *Salmonella enterica*. In *S. enterica*, a pathogen of humans and livestock, lineage formation plays roles in host colonization, biofilm formation, motility, bacteriophage resistance and adaptive resistance to antibiotics, among others (see Table 2 in (Fernández-Fernández et al., 2022) for a summary of the role associated with lineage formation in *Salmonella*) (Clegg et al., 1996; Cummings et al., 2006; Broadbent et al., 2010; Saini et al., 2010; Ahmad et al., 2011; Zorraquino et al., 2013; García-Pastor et al., 2018; Liu et al., 2019; Sánchez-Romero and Casadesús, 2021a).

Cell sorting, also commonly referred to as cell separation or cell isolation, is a procedure used to isolate one or more specific cell populations from a heterogeneous mixture of cells. Bacterial subpopulations are detectable in the laboratory, and may be formed equally or more frequent in natural environments (Brehm-Stecher and Johnson, 2004). The use of isolated cells to perform experiments allows scientists to assuredly answer specific questions by diminishing interference from other cell types within the population (Davey and Kell, 1996; Mattanovich and Borth, 2006; Robinson, 2022). Isolated cells have many applications within life science research, allowing scientists to describe the distribution of cellular properties within a clonal population. In this review, we describe several single-cell approaches of how scientists use isolated cells in different subjects to analyze *Salmonella* lineage-specific traits. We expose the importance of single-cell technology in revealing invaluable information about the heterogeneous composition of bacterial populations.

## 2 Cell separation procedures

### 2.1 Parameters to consider for cell isolation procedures

Several parameters must be taken into account when evaluating the performance of a cell separation procedure. The main variable parameters to be considered are yield, purity, recovery, and viability. Moreover, cost and speed should also be contemplated (Davies, 2012). While yield refers to the total number of recovered target cells, purity refers to the proportion of desired cells in the final isolated cell fraction, and is generally expressed as a percentage of total live cells. Apart from that, recovery refers to the proportion of desired cells isolated to the desired cells available in the starting sample. This parameter gives information about how many of the desired cells have been lost through the cell separation method. Although this is not always the case, purity and recovery tend to be inversely correlated. A balance between purity and recovery is needed for the isolation of highly purified cells with the number of target cells. The importance of the different functional parameters will depend on the specific experimental purposes. For instance, when sufficient material is available, yield may be less important and high purity may be essential. Hence, special focus should be given to the election of cell sorting parameters and strategy or method, according to the identified goals.

### 2.2 Types of cell separation methods

**Figure 1:**
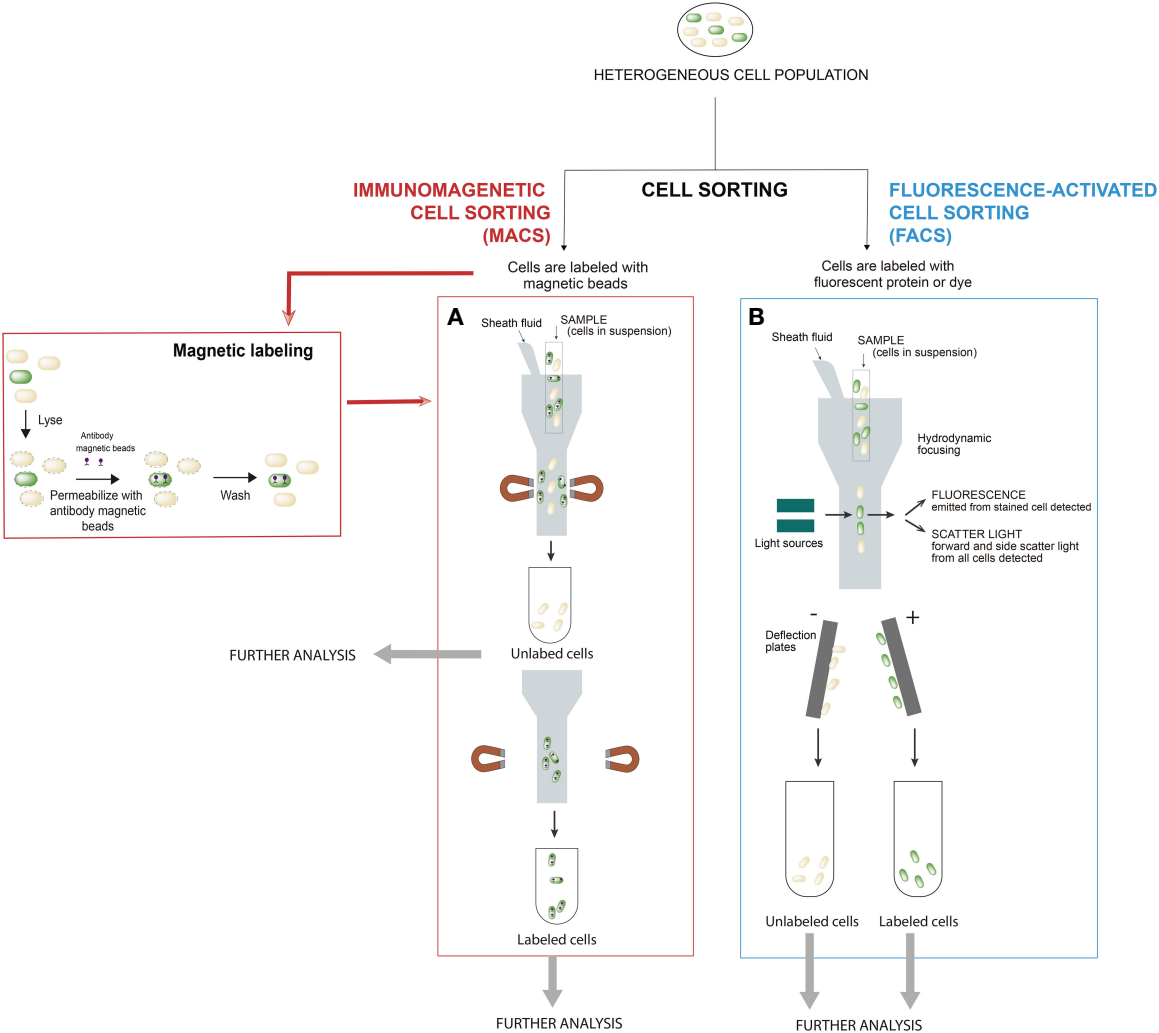
Most common cell sorting techniques for screening microbial populations: **(A)** Immunomagnetic cell sorting (MACS) and **(B)** Fluorescence-activated cell sorting (FACS). In the procedures for bacterial cell handling during separation by cell sorting, cells are treated according to the respective protocols related to the type of cell sorting to be performed. In MACS, cells are magnetically labeled with microbeads and FACS requires that cells are stained with either a dye(s) or fluorescent protein(s). In MACS, firstly, labeled cells with magnetic beads are retained in the system, while unlabeled cells pass through (negative cell fraction). Secondly, the magnet is removed from the sample and the target magnetic-bead-labeled cells are eluted as the enriched, positively selected cell fraction. In FACS, positive and negative cells for fluorescence are separated simultaneously. Modified from (Fernández-Fernández et al., 2022).

A great variety of cell separation methods are available to isolate cells from complex biological samples, each with its own advantages and drawbacks. The cell separation method you choose depends on what the use of the isolated cells is, and the choice may involve a trade-off. Common characteristics used to isolate cells include cell size, cell density, cell shape, and protein expression. The most usual cell separation techniques include immunomagnetic cell sorting (MACS) and fluorescence-activated cell sorting (FACS) (**Figure 1**). Other cell separation methods, less frequently used for bacterial populations, are density gradient centrifugation and immunodensity cell isolation for applications such as separation of plasma from blood cells or fractionation of peripheral blood mononuclear cells.

#### 2.2.1 Immunomagnetic cell sorting

Immunomagnetic cell separation (MACS) is a technique that relies on magnetic labeling of cells prior to separation in a magnetic field (Miltenyi et al., 1990). Magnetic particles are bound to specific protein on the target cells by different molecules (antibodies, enzymes, lectins, etc.). The sample is then placed in a magnetic field, bringing the labeled cells with them. As unlabeled cells are not retained by the electromagnetic field, a physical separation between target and non-target cells is created within the sample (**Figure 1A**). This technique is one of the most used methods to purify specific cells of heterogeneous population due to its simplicity, less time consuming (fast and easy protocols), low equipment cost and automatization. Although many cells can be isolated at once, it lacks the sensitivity and the cell specificity provided by other fluorescence-based techniques (Fernández-Fernández et al., 2022).

#### 2.2.2 Fluorescence-activated cell sorting

A basic and useful procedure for the isolation and characterization of subpopulations in a bacterial culture is the use of reports that rely on fluorescence. There is an extensive catalog of fluorescent proteins and the choice of fluorochrome depends on both the experiment to be done and the instrument used to measure the signal (Fernández-Fernández et al., 2022). Fluorescence-activated cell sorting (FACS) is a method that uses flow cytometry and fluorescent probes to sort heterogeneous mixtures of cells (Bonner et al., 1972). Flow cytometer focuses the cell suspension into a uniform stream of single cells. This stream is then passed through a set of lasers that excites the fluorophores, causing light scattering and fluorescent emissions. Based on the wavelengths produced by laser excitation, the cytometer collects information about the different types of cells within the sample (**Figure 1B**). Although immunomagnetic cell sorting is a much faster and simpler procedure than FACS, FACS is often the preferred cell isolation method for the separation of bacterial lineages. In FACS, the principle of gating is crucial, as it enables a subset of data/cells from the larger data set to be selected and analyzed. Hence, FACS has the ability to sort single cells and isolate cells based on intracellular markers (e.g. GFP), on surface marker expression levels (e.g. fluorescent dyes) and sort complex cell types with multiple markers at higher purity (Fernández-Fernández et al., 2022).

#### 2.2.3 Other cell isolation techniques

Cell sorting based on immunomagnetic and fluorescence are the strategies more commonly used to resolve the properties of single cells and to characterize bacterial subpopulations.

Despite their widespread use, these methodologies might have limitations. The emergence of multi-omics methods has put an emphasis on the importance of sample preparation. Cell sorters work at high pressure that could cause cellular damage, causing decreased viability, reduced growth, or cellular damage after sorting. Rather than using high pressure fluidics for sorting cells, microfluidic technologies, known as ´lab-on-a-chip´, are developing a category of cell separation methods which designs devices to manipulate fluids on a microscopic level to facilitate single-cell isolation without experiencing shear stress (Shields et al., 2015).

The use of fluorescence-based techniques with FACS or MACS are extremely effective in the capability of differentiating heterogenous populations. Nonetheless, they fail to provide information about the localization of fluorescence in cells and the tracking of dynamic cell behaviors. In this sense, imaging flow cytometry (IFC) combines the high speed and data acquisition of flow cytometry and features of fluorescence microscopy with data-processing algorithms (Basiji et al., 2007). Continual improvements to IFC have been developed, however, so far, its applicability for sorting of bacterial cells has not yet been reported.

Other cell separation technologies, including density gradient centrifugation, immunodensity cell separation, sedimentation, adhesion, aptamer-based cell isolation, optical tweezers, and laser capture microdissection, have been successfully used for a variety of biological applications (Tomlinson et al., 2013). Furthermore, several approaches are being developed to address the limitations of existing cell sorting technologies, combining different technologies. For instance, Tewari Kumar et al. demonstrated the integration of optical tweezers with microfluidic technologies for trapping *Salmonella* captured on magnetic beads (Tewari Kumar et al., 2020). This methodology allows spatially organize and select bacteria at single-cell level for further downstream analysis.

## 3 Cell sorting applications for analysis of *Salmonella* lineage-specific traits

**Figure 2:**
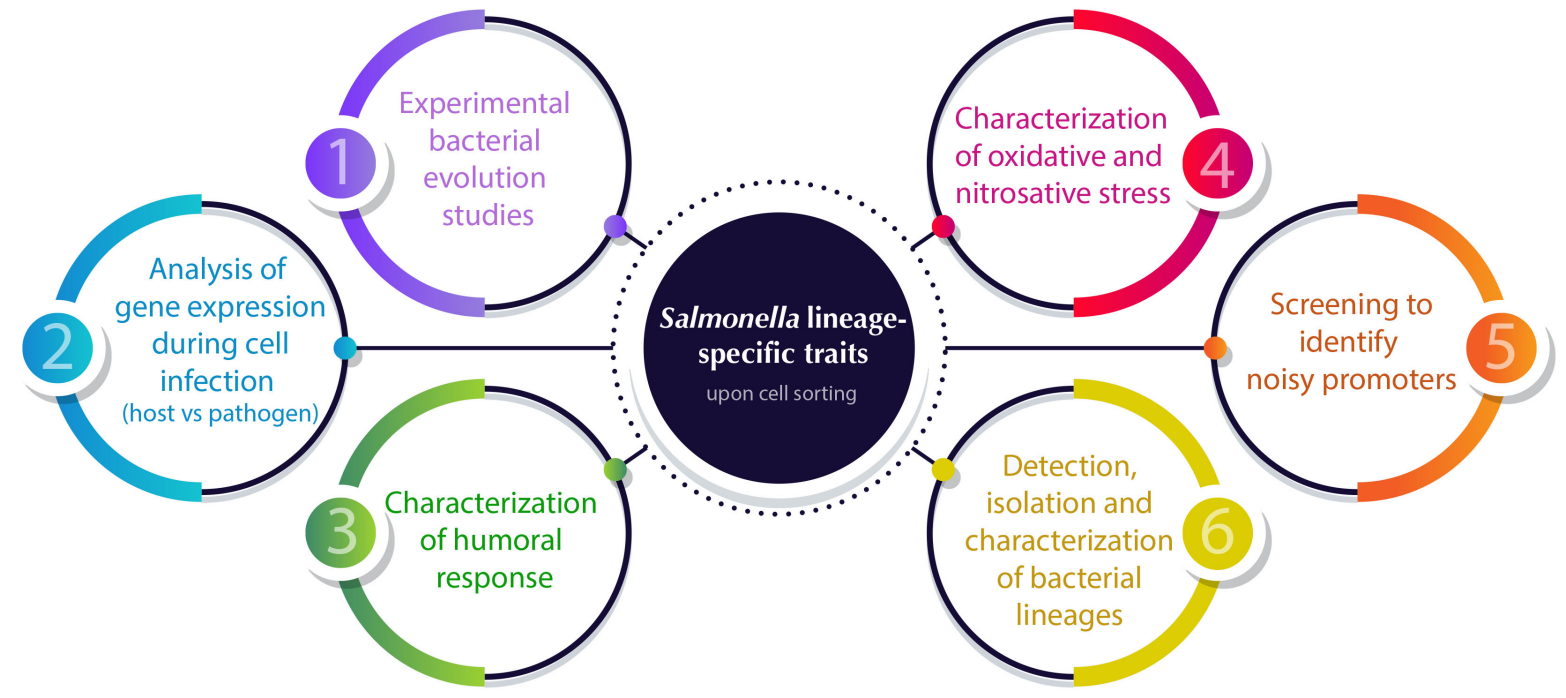
Overview of cell sorting applications for analyzing *Salmonella* lineage-specific traits to solve the structure of bacterial populations. Numbers indicate the sections in which the applications are described in detail.

In the next section, we will describe several single-cell approaches that have recently been applied to describe the distribution of cellular properties within a *Salmonella* clonal population with different purposes (**Figure 2**).

### 3.1 Experimental studies of bacterial evolution

Microbial evolutionary experiments gained accuracy using whole genome sequencing. Nevertheless, bacterial clonal populations showed phenotypic heterogeneity, consequently, their study in terms of evolution entails a specific analysis of subpopulations. The appearance of mutations occurs at different points through the evolution process and could be the responsible of shaping bacterial lineages. Bawn et al. combined bacterial isolation by FACS with single-cell genome sequencing to obtain the appearance of genome diversity within the bacterial population in response to exposure to antibiotic stress (Bawn et al., 2022). Bawn et al. used a hypermutator strain of *Salmonella* and compared it with wild-type (WT) *Salmonella enterica* (serovar Enteriditis) under ciprofloxacin exposure. They concluded that the number of SNPs in the hypermutator strain in the presence of ciprofloxacin is significantly higher compared to that of the WT strain. Moreover, analysis of the phylogeny and population structure of these strains showed the presence of 7 clades. The main difference is that WT is located within clade 7 and the hypermutator strain evolved, determining the other 6 groups. Three of these groups were ciprofloxacin positive with changes in genes previously identified as related to antibiotic resistance (Bawn et al., 2022). The authors affirmed that this structural resolution of different subpopulations could not be identified from the whole genome sequencing of the bulk culture and highlighted the importance of high-throughput single-cell sequencing to improve experimental studies of bacterial evolution.

### 3.2 Gene expression analysis and bacterial and host factors involved in the infection process

In addition to environmental adaptation, bacteria and host cells adjust gene expression during the infection process. Transcriptomics of both the pathogen and the host, separately, gives us only a side of the whole story. Dual RNA sequencing is a protocol that analyzes gene expression changes in both the model pathogen *Salmonella* serovar Typhimurium and the human cells at the same time (Westermann et al., 2012; Westermann et al., 2016). In this protocol, the authors used FACS technology to enrich HeLa cells infected with *Salmonella* and isolate their RNAs, giving the dynamic RNA expression landscape of both a bacterial pathogen and its eukaryotic host during the course of infection (Westermann et al., 2016). This method has allowed the authors to study the real host response to bacterial infection. Prior to dual RNA sequencing, host response to pathogen infection is masked in bulk expression analyses by non-infected cells since an enrichment step is not carried out.

Intracellular bacterial pathogens can exhibit large heterogeneity in intracellular host proliferation. (Saliba et al., 2016) combined cell sorting and single-cell RNA-sequencing analysis to study the macrophage response to different intracellular states of *Salmonella enterica* serovar Typhimurium. They studied the transcriptomes of individual infected macrophages harbouring either growing or non-growing bacteria. The results showed that gene expression heterogeneity among macrophages creates different cellular environments for *Salmonella* to either facilitate immune evasion with a growth arrest or escape intracellular antimicrobial activity and exploit its host for maximal proliferation.

Microscopy of liver sections infected with *Salmonella* revealed the existence of two different types of macrophages based on their infection level: weakly and highly infected macrophages (Sheppard et al., 2003). Weakly infected macrophages contain one or two *Salmonella* cells and are predominant over the highly infected ones, which present more than two *Salmonella* cells. Due to their abundance, weakly infected macrophages have been considered relevant to the infection process. The importance of the small subpopulation of highly infected host cells remained unclear since the comparison between different host cells required a very sensitive method to separate these subpopulations (Sheppard et al., 2003). Thöne et al. undertook a rapid technique, based on FACS technology, to characterize differentially *Salmonella*-infected host cell subpopulations from infected mouse spleen and to identify bacterial and host factors that control *Salmonella* load and proliferation *in vivo*. The results highlighted the importance of highly infected macrophages, showing that highly infected host cells are not more activated than weakly infected cells in mouse spleen (Thöne et al., 2007).

Pathogenic bacteria are able to adapt to heterogeneous host environments by modifying the expression of their genes. Consequently, analysis of gene expression pre- and post- infection (within and outside hosts) may be critical to understand the infection process (Chiang et al., 1999). Host environments are too complex and dynamic to be accurately reproduced in the laboratory (Rediers et al., 2005). Bumann & Valdivia (2007) overcame that drawback by developing an approach that combines differential fluorescence induction (DFI) with FACS (Bumann and Valdivia, 2007). Following their methodology, animals are infected with a library of bacteria carrying random insertion of *gfp* transcriptional gene fusions. Fluorescent bacteria can be isolated directly from infected cells or tissues using FACS (Valdivia and Ramakrishnan, 2000). Based on their fluorescence, bacteria expressing different GFP intensities can be well distinguished. After separation by FACS, gene expression analysis in recovered cells can be performed. Indeed, this technology can be complemented by adding an optional step: fluorescent cells recovered by FACS can be grown in laboratory media. From laboratory media, non-fluorescent bacteria can be separated by FACS and be used to re-infect animals. Once inside the animals, fluorescent cells are again separated and recovered from tissues using FACS. The last group of sorted cells contained transcriptionally active promoters within host tissues that are inactive outside hosts (Bumann and Valdivia, 2007).

### 3.3 Noise in gene expression associated with host-pathogen interactions

The occurrence of different behaviors among individual cells in microbial clonal populations has been documented more than two decades ago (Elowitz et al., 2002). Cell-to-cell fluctuations in gene expression within an isogenic culture as a consequence of stochasticity or noise can be the origin of phenotypic variation (Eldar and Elowitz, 2010). To describe the molecular causes and biological functions of the phenotypic noise in *S. enterica* serovar Typhimurium, in 2008 Freed et al. developed a method for the identification of genes displaying stochastic expression (Freed et al., 2008). This method consists of a genomic plasmid library of promoters controlling GFP expression and cloned in *Salmonella*. *Salmonella* populations containing this library were cultivated in exponential phase and sorted by FACS for seven rounds of selection. The system of selection contained two fluctuation steps in each round: first, an enrichment of high GFP expression and second, an enrichment of low fluorescence. After rounds of fluctuating selection, the *Salmonella* population was enriched in promoters with a high average noise in gene expression. Sequencing analysis of these promoters showed that genes that exhibit the highest levels of variation control in the expression are related to flagellum synthesis, virulence and host-pathogen interactions. Freed et al. suggested that different subpopulations of *Salmonella* with phenotypic variation in infection-associated genes could be beneficial for labor division during cell invasion or gut competition (Freed et al., 2008). This work describes a simple method to select ´noisy´ promoters that could be applied to other unicellular organisms and under an enormous variety of conditions.

### 3.4 Characterization of humoral response to antigenic structures such as flagellin in chicken

Flagella appears to play an essential role prior to *Salmonella* infection (Elhadad et al., 2015). The *fliC* gene encodes one globular protein exposed in the flagellum, one of the principal targets of the immune system. Wang et al. have analyzed the *in vivo* humoral response in chickens triggered by FliC of *S.* Enteriditis (Wang et al., 2016). First, a library of FliC antigens with a variety of sizes of cloned *fliC* gene was constructed in yeast. Upon induction of this library, the expression of a great battery of FliC antigens on the surface of yeast was achieved and then the cells were labeled by using chicken antisera against *S.* Enteriditis. Several rounds of cell sorting were performed by FACS in order to increase the FliC antigenic library. To identify these FliC antigens, plasmid extraction was performed, cloned into *E. coli*, and then sequenced. When these sequences were mapped to the *fliC* gene, they formed three different domains able to elicit strong humoral responses *in vivo*. These domains showed a high reaction against the antisera and were then chosen to immunize chickens. Finally, *in vivo* bacterial colonization was measured in different organs of chickens immunized with the FliC selected domains and significant reductions in bacterial colonization were found in these chickens compared to the control. This work will facilitate our better understanding of humoral responses after infection by *S*. Enteritidis.

Effective vaccines should appeal all types of immune response, including the innate immune response, the adaptive immune response, and the immune memory. Flow cytometry coupled with cell sorting might be very useful to identify potential targets for vaccines against *Salmonella* by revealing how the immune system responds to different strategies.

### 3.5 Characterization of oxidative and nitrosative stress on *Salmonella* during infection in mouse

One of the host defense mechanisms against bacterial infection is the production of reactive oxygen and nitrogen species, but its mode of action is still in debate (Mastroeni et al., 2000; Aussel et al., 2011). Measurement of these reactive species as target cells for defense is not accurate if made as a whole. To solve this problem, single-cell measurements combined with cell sorting analyses have been performed by (Burton et al., 2014) to analyze how reactive oxygen and nitrogen species fight *Salmonella* infection in mice. They have used a system to discriminate total cells and live cells. This procedure is based on the detection of total *Salmonella* with a LPS antibody and live *Salmonella* with an intracellular fluorescent protein (which is retained intracellularly only in live cells) (Barat et al., 2012). After 4 days of infection, most *Salmonella* found in neutrophils and inflammatory monocytes in inflammatory lesions were dead, while many live *Salmonella* cells were discovered after infection of mice mutated into one of the NADPH oxidase subunits. Furthermore, the analysis of resident macrophages did not show any differences between mice deficient in NADPH oxidase and wild type. Since the enzyme NADPH oxidase produces reactive oxygen species (ROS), these results involve that *Salmonella* infection is controlled in neutrophils and inflammatory monocytes by the action of ROS, but this mechanism does not act in macrophages. The combination of these results with a computational model allows to conclude that ROS have a clear bactericidal effect during *Salmonella* infection in neutrophils and inflammatory monocytes (Burton et al., 2014).

*Salmonella* does not appear to be affected by the effect of NADPH oxidase in macrophages. To investigate whether oxidative stress controls *Salmonella* infection in macrophages, (Burton et al.) constructed an episomal *katGp-gfpOVA* fusion. OxyR is a transcriptional factor related to oxidative stress, and when it binds to H_2_O_2_, it activates the *katGp* promoter. Using this approach, they detected by flow cytometry a heterogeneous population containing different activities for the *katGp* promoter, GFP^dim^ and GFP^bright^. These results showed the variety of levels of oxidative stress in a population of *Salmonella* cells, which could reflect different exposure to ROS. To test this hypothesis, Burton et al. take advantage of cell sorting to isolate cells from GFP^dim^ and GFP^bright^ subpopulations in different mutants constructed in *Salmonella* and to infect mice already pre-infected by non-fluorescent *Salmonella* cells (Burton et al., 2014). The results showed an increase in GFP fluorescence in the sorted subpopulation during the early infection, displaying a transient ROS exposure after cell infection. The analysis of the proteome of these two subpopulations of *Salmonella* (GFP^dim^ and GFP^bright^) showed that GFP^bright^ contains upregulated the oxidative stress response system.

As reactive nitrogen species are also involved in the response against infections, the authors replaced the OxyR promoter controlling GFP with the NsrR promoter, the activity of which is triggered in the presence of reactive nitrogen species. In this case, GFP expression showed bimodal activity showing a different proportion of GFP^bright^ in a subpopulation between individuals and was absent in a mutant lacking iNOS, an enzyme that produces reactive nitrogen species. Similar approach to ROS species was performed using FACS to isolate and purify *Salmonella* cells from GFP^bright^ and GFP^dim^ subpopulations and compare the proteomes of both subpopulations (Burton et al., 2014). Proteomic analysis of single cells showed up-regulation of three targets of the NsrR transcription factor related to reactive nitrogen species.

Burton et al. analyzed both oxidative and nitrosative stresses in the same cells. To test it, they used a system with two biosensors: ROS biosensor was detected by RFP (Red Fluorescent Protein) expression and NOS biosensor by GFP (Burton et al., 2014). When both biosensors were tested simultaneously in the infected spleen, four different subpopulations were found by combination of RFP/GFP in its two dim/bright modalities. This approach reveals the independence of both responses in *Salmonella* after infection.

### 3.6 Detection, separation and characterization of bacterial lineages

#### 3.6.1 Infective organisms

Conventional detection methods used to identify *Salmonella* are well established, but require numerous and complex steps. Biosensors, those devices with a biological component to detect an analyte and an element to produce a signal, are a rapid and simple pathogen detection approaches. These designs are based on a binding reaction between a bioligand (enzyme, antibodies, cells, and nucleic acids) immobilized on the surface of the biosensor and pathogen cell (Torres-Chavolla and Alocilja, 2009). The specificity and sensitivity of these biosensors depend on the affinity of ligands for the pathogen (Joshi et al., 2009). Aptamers are specific and short DNA or RNA sequences that present great potential as bioligands, because they are small, easy, and cheap to synthesize (Tombelli et al., 2005).

Whole-cell SELEX (Systematic Evolution of Ligands by EXponential enrichment) method can be used to produce aptamers that bind to the surfaces of living pathogens. This method relies on exponential enrichment process that ensures the affinity between aptamers and pathogen cells (Daniels et al., 2003). During the whole-cell SELEX process, a random DNA library is incubated with the bacterial cells of interest. Unbound DNA is removed by centrifugation and bound DNA is released from bacteria and amplified for subsequent rounds of binding. After several binding-rounds, different aptamers are maintained in the DNA pool. As whole-cell SELEX could select aptamers with good affinity but with poor specificity for the target, FACS can be used to screen and select aptamers that are specific to the target and, in this way, to increase the efficacy of the process (Hamula et al., 2011). Aptamer candidates are fluorescently labeled and incubated with the target bacterial cells. Since the pool contains different aptamer sequences, those aptamers that bind to the bacteria would be expected to be those with the highest specificity and affinity for their targets. By using FACS, it is possible to separate fluorescent cells, that is, those cells showing the most affine aptamers from the rest of the aptamers present in the pool. A remarkable advantage of the use of FACS is the possibility of samples preservation after sorting. Thereby, aptamers can be recovered and analyzed. Moon et al. reported a method that combines whole-cell SELEX approach with FACS to select aptamers with the highest affinity and specificity for *S. enterica* serovar Typhimurium (Moon et al., 2013). In this study, SELEX was used to select aptamers present in the DNA pool after 10 selection-rounds, and, subsequently, the selected aptamers were fuorescently labeled and incubated with live *Salmonella* cells. FACS was utilized to separate those cells that have bound the highly enriched oligonucleic acid pool, in other words, aptamers with the highest affinity. Consequently, a total of 12 aptamers were chosen for subsequent analysis. These aptamers were released for further analysis, including DNA amplification and sequencing.

#### 3.6.2 Mutants tolerant to biocides

The use of biocides is crucial to prevent bacterial infections, and its application is gaining attention because of the increase in antibiotic resistance (Russell, 2003). Due to the fact that many biocides share molecular targets in bacteria with antibiotics, bacteria that survive to a low dose of biocide are more likely to be resistant to antibiotics (Karatzas et al., 2008). Hence, the study of mechanisms involved in biocide resistance can be useful in fighting against the antimicrobial resistance.

Whitehead et al. employed flow cytometry coupled to cell sorting to analyze *Salmonella enterica* cells which survive to biocide exposure (Whitehead et al., 2011). Despite the low frequency of biocide-resistant cells, FACS allowed their physical isolation and an antibiotic resistance profile of the sorted biocide resistant cells was carried out. These results showed that the exposure to different biocide concentrations produces changes in susceptibility of *Salmonella* to different antibiotics. Efflux activity in sorted cells was investigated, revealing that mutants recovered from in-use doses of biocide presented an increase in efflux activity (Whitehead et al., 2011). This study demonstrated the influence of the over-expression of the AcrEF efflux pump on the resistance to biocides and antibiotics.

#### 3.6.3 Acid-inducible promoter

The entry of *Salmonella* Typhimurium into mammalian cells leads to the formation of phagosomes (Francis et al., 1993; Alpuche-Aranda et al., 1994). Acidic pH inside phagosomes seems to be important for bacterial survival and proliferation in murine macrophages (Rathman et al., 1996). A transcriptomic analysis of *Salmonella* within macrophages showed that acid shock proteins were the most induced gene intracellularly (Eriksson et al., 2003). In other words, survival of *Salmonella* Typhimurium in the intracellular environment may depend on the transcription of a set of pH-regulated genes that could protect *Salmonella* from phagocyte’s killing mechanisms (Valdivia and Falkow, 1996). For this reason, the study of acid-inducible gene expression in phagosomes might elucidate the role of low pH as an intracellular inducer of bacterial gene expression (Valdivia and Falkow, 1996; Viala et al., 2011)

Valdivia & Falkow investigated this issue by combining DFI (explained above) and FACS. Cells of *S.* Typhimurium that carried random fusions of *gfp* were subjected to low pH, and fluorescent bacteria were collected by FACS (Valdivia and Falkow, 1996). To distinguish fluorescence between constitutive and pH-inducible gene expression, collected cells were exposed to neutral pH. The highly sensitivity and processivity of FACS provided a powerful approach to isolate inducible genes based on small differences in expression. Valdivia & Falkow characterized eight acid-inducible promoters in detail and quantified their expressions after low pH exposure (Valdivia and Falkow, 1996). Comparison between these two conditions revealed that some promoters exhibited higher expression within macrophages than in acid-shock, thus suggesting that additional inducer signals may exist within macrophages. Besides, kinetics measurements of gene expression performed by FACS allowed to determine the point of maximum expression of these genes that occurs after 4-5 hours of bacterial entry into a macrophage (Valdivia and Falkow, 1996).

#### 3.6.4 Bacterial ghost

Bacterial ghosts are empty bacterial cell envelopes of Gram-negative bacteria. They are considered as candidate vaccines (Eko et al., 1999; Lubitz et al., 1999) due to their ability to activate both cellular and humoral immune response in animals (Eko et al., 2000; Hensel et al., 2000). Ghosts’ production consists of cloning and expressing of the lysis gene *E*, which triggers the formation of a transmembrane tunnel. Due to the high osmotic pressure inside the cell, the bacteria content is expelled into the surrounding media, leaving the bacteria cell envelope empty (Witte et al., 1992; Schön et al., 1995).

In ghost preparations of *E. coli* and *Salmonella*, some non-lysed cells remain. To obtain pure ghost preparations, these viable bacteria must be eliminated (Haidinger et al., 2001). Methods applied to achieve this goal should ensure ghost features preservation and do not imply large loss of ghost material. In this context, *gfp* expression followed by FACS purification can aid to differentiate viable bacteria cells from ghosts. Haidinger et al. described a procedure that considerably improves the purity of ghost preparations, using a strain of *E. coli* with a plasmid encoding *gfp*. First, they induce *gfp* expression and second, the protein E. Lysed cells expel all their cytoplasmatic content outside the cell envelope (Haidinger et al., 2001). This implies that the previously synthesized GFP protein is also ejected into the surrounding media, leading to loss of fluorescence in ghosts. Hence, while ghosts are non-fluorescent, non-lysed cells remain highly fluorescent. By flow cytometry, the non-lysed cells can be well distinguished from ghosts and the sorting process permits the recovery of ghosts.

#### 3.6.5 Lineages differing in antibiotic susceptibility

Bacterial subpopulations can exhibit different susceptibilities to a particular antibiotic. Furthermore, cellular responses to a given antimicrobial agent are often heterogeneous and led to the formation of different subpopulations that manage to survive in the presence of antibiotics through different mechanisms, such as persistence, heteroresistance, and/or tolerance (Balaban et al., 2004; El-Halfawy and Valvano, 2015; Hjort et al., 2016; Fisher et al., 2017; Nicoloff et al., 2019). In studies that employ bulk cultures, quantitative phenotypes are usually averages obtained for a given parameter at the population level. This approach is perfectly appropriate for the population as whole but does not permit the detection of subpopulations with different phenotypes from that of the main subpopulation. The formation of heteroresistant, persistent and/or tolerant bacterial cell might be the cause of failure treatment in hospitals when it is assumed that the susceptibility of bacterial pathogen to a given antibiotic is reflected by average values. Elucidating these subpopulations or rare cells can be technically challenging.

In this regard, flow cytometric cell sorting contributes to characterizing *Salmonella* bacterial lineages that show different antimicrobial susceptibilities, thus clarifying aspects of antibiotic resistance. For instance, Sánchez-Romero & Casadesús used cell sorting to separate *Salmonella* lineages according to their expression of *ompC* porin in two subpopulations (cells with low and high expression of *ompC*) (Sánchez-Romero and Casadesús, 2014). After sorting, these cells were recovered and characterized regarding their resistance to kanamycin. This investigation correlates low expression of *ompC* with higher resistance to kanamycin and reveals that kanamycin resistance can be accomplished by non-mutational mechanisms. Similar studies used FACS to sort cells based on reporters for persistence-implicated genes and then utilized those cells for comparison to their phenotypes (Helaine et al., 2010; Ramos-Marquès et al., 2017; Luk et al., 2021). Luk et al. identified a dormant *S*. Typhimurium subpopulation in infected epithelial cells that are phenotypically distinct from that observed in macrophages and fibroblasts. This work revealed the potential of fluorescent reporters and cell sorting in bacterial characterization of its persistence. Ramos-Marquès et al. demonstrated that *S*. Typhimurium attenuates NF-κB signaling in fibroblasts, an effect only perceptible when subpopulations are analyzed separately. Heleine et al. reported the development of a reporter system based on fluorescence dilution that enables direct quantification of the replication dynamics of *S*. Typhimurium in murine macrophages at single-cell level, and to identifying and characterizing nonreplicating bacteria associated with chronic or latent infection.

#### 3.6.6 Lineages differing in virulence gene expression

*Salmonella enterica* pathogenicity island 1 (SPI-1) and *Salmonella enterica* pathogenicity island 2 (SPI-2) are gene clusters that encode type III secretions systems and effectors implicated in interactions between *Salmonella* and its hosts. Both SPI-1 and SPI-2 undergo bistable expression that results in the formation of *Salmonella* ON and OFF lineages (Hautefort et al., 2003; Sturm et al., 2011; Sánchez-Romero and Casadesús, 2018).

The involvement of SPI-1^ON^ and SPI-1^OFF^ in epithelial cell invasion was investigated by FACS. Cell sorting followed by epithelial cell invasion assays revealed that both subpopulations are required for optimal invasion (Sánchez-Romero and Casadesús, 2018). Moreover, the capacity of FACS to distinguish simultaneously numerous subpopulations enabled to test the expression of SPI-1 together with the flagellar (Flag) regulon. Based on the expression patterns, four subpopulations were identified: SPI-1^ON^ Flag^ON^, SPI-1^ON^ Flag^OFF^, SPI-1^OFF^ Flag^ON^ and SPI-1^OFF^ Flag^OFF^. These four subpopulations were sorted by FACS and recovered for subsequent analysis. This study showed that an independent switch between SPI-1 and Flag regulation is present and Flag^OFF^ cells may contribute to optimal invasion (Sánchez-Romero and Casadesús, 2021a).

#### 3.6.7 Lineages differing in flagellar and fimbrial gene expression

*Salmonella* fimbriae are expressed in the mammalian intestine and mediate adhesion to animal tissues (Humphries et al., 2003; Baumler et al., 2011). Expression of fimbriae is often subjected to phase variation and entails the formation of fimbriated and non-fimbriated lineages. Flow cytometry has been crucial to a better understanding of the physiological significance of fimbriae bimodal expression (Cummings et al., 2006; Saini et al., 2010; Koirala et al., 2014; Stewart and Cookson, 2014; Zarkani et al., 2020; Sánchez-Romero and Casadesús, 2021a).

In *Salmonella enterica*, the *std* locus encodes fimbriae that permit adhesion to epithelial cells in the large intestine. The expression of *std* is subjected to phase variation and leads to the formation of a Std^OFF^ (non-fimbriated) and Std^ON^ (fimbriated) subpopulations. Despite small fraction of OFF lineage (0.3-3%) under laboratory conditions, FACS and MACS allowed for sorting Std^OFF^ and Std^ON^ subpopulations (García-Pastor et al., 2018; García-Pastor et al., 2019). Further analysis on these cells indicates that both subpopulations may differ not only in the possession of Std fimbriae but also in additional phenotypic traits such as motility, which is lower in the Std^ON^ subpopulation (García-Pastor et al., 2018).

## 4 Conclusions and future perspectives

Traditionally, the formation of cell lineages has been considered a eukaryotic phenomenon. Nevertheless, during the last years, the capacity of a bacterial population to produce phenotypic cell variants has been explored and it has revealed a distinct conception of the structure of bacterial populations. Cell sorting technology has demonstrated its potential to expand our understanding of the behavior of bacterial populations. In the years to come, it is expected that the advancement of the single-cell technology will become the use of cell sorting as a routine tool in the microbiological laboratories.

## Author contributions

MS-R and JC: conceptualization, investigation, administration and funding acquisition. RF-F, RL-I and MS-R: original draft preparation. RF-F, RL-I and MS-R: review and editing. All the authors contributed to the article and all, except JC who passed away on August, approved the submitted version.
